# More to B: the growing evidence to inform targeting B cells in scleroderma

**DOI:** 10.1093/rheumatology/keac677

**Published:** 2022-12-05

**Authors:** Silvia Laura Bosello, Edward M Vital, Francesco Del Galdo

**Affiliations:** UOC di Reumatologia, Università Cattolica del Sacro Cuore, Fondazione Policlinico Universitario A. Gemelli, IRCCS, Rome, Italy; Leeds Institute of Rheumatic and Musculoskeletal Medicine, University of Leeds, Leeds, UK; NIHR Leeds Biomedical Research Centre, Leeds Teaching Hospitals NHS Trust, Leeds, UK; Leeds Institute of Rheumatic and Musculoskeletal Medicine, University of Leeds, Leeds, UK; NIHR Leeds Biomedical Research Centre, Leeds Teaching Hospitals NHS Trust, Leeds, UK

**This editorial refers to ‘The role of B cells in the pathogenesis of systemic sclerosis: an update’, by Sakkas *et al.* 2023;62: 1780–6.**

SSc carries the highest morbidity and mortality across CTD. Effective treatment and clinical control of the disease remain challenging. Currently available treatment approaches slow disease progression while recent trials have informed new treatment strategies to help patients. In this issue of *Rheumatology*, Sakkas *et al.* share an interesting review of the new evidence supporting targeting B cells beyond the ‘classic’ approaches employed so far. This is particularly timely since in the past 6 months a new wave of strong clinical level evidence has supported the efficacy of B cell targeting in SSc skin and lung involvement.

Although the pathogenesis of SSc is incompletely understood, three pathogenetic mechanisms have been identified as crucial: endothelial abnormalities with vascular damage, imbalance of immune system with inflammatory burden, and fibroblast activation with extensive extracellular matrix deposition and fibrosis.

A dynamic interplay between these mechanisms in different disease stages is the core of the most credited pathophysiological model of SSc. Understanding the dominant pathogenic driver of cutaneous and other organ involvement in each stage of the disease could guide selection of appropriate therapeutic interventions within the heterogeneity and complexity of the spectrum of clinical manifestations in SSc.

Sakkas and colleagues reviewed the crucial role of B cells and underlined the recent evidence on B cell abnormalities in SSc disease [1, 2]. Multi-omics evidence strongly supports the role of B cells both in the early stage of SSc and during progressive organ involvement.

There are extensive reports of B cells in biopsies of organs affected by SSc, such as skin, lung and gastrointestinal tract. Multi-omic approaches have indicated their activated phenotype as well as their roles beyond being the source of autoantibody-secreting cells, including inflammatory and profibrotic cytokine secretion, loss of regulatory properties, and through crosstalk with numerous other cells including endothelial cells, vascular smooth muscle cells, T cells, dendritic cells, other immune cells and, finally, fibroblasts.

The role of SSc-specific antibodies in predicting progression to disease in patients with Raynaud’s phenomenon has been recently validated in a large multicentre European cohort [3]. Further, a multiomic approach to diffuse SSc molecular heterogeneity has indicated that specific autoantibodies such as antitopoisomerase-1 and anti-RNA polymerase III were linked to distinct serum protein markers of fibrosis over time as well as divergent gene expression profiles [4]. Nevertheless, it is not clear whether they play a major and direct role in SSc pathogenesis. Sakkas *et al.* review a recent study revealing that Topo I-autoreactive B cells produce a variety of cytokines depending on the affinity of their B cell receptor to Topo-I. In particular, B cells with high affinity for Topo-I showed a profibrotic profile due to proinflammatory cytokine production and proinflammatory T_H_17 polarization, while B cells with low affinity for Topo-I were antifibrotic due to anti-inflammatory cytokine production and induction of regulatory T cells.

These findings further support the notion that restoring B cell homeostasis in scleroderma with specific intervention could promote disease control. In this context the use of chimeric antigen receptor-T (Car-T) cells targeting CD19 or B cell receptor with high affinity for anti-Topo-I might be a future innovative therapeutic strategy for SSc as it has been recently proposed for refractory SLE [5].

Parallel to this line of evidence, in SSc there is an imbalance in effector and regulatory functions of B cells due to an increase in B cell secretion of proinflammatory cytokine IL-6 and autoantibodies, and a reduction of secretion of anti-inflammatory cytokine IL-10 [1]. This disrupted homeostasis and hyperactivation encountered in SSc B cells probably influences many of their regulatory immune functions and favours the emergence of autoimmune reactions [2].

Considering the growing evidence supporting the role of B cells in SSc, rituximab (RTX) has been used in everyday clinical practice although not licensed in Europe or the USA for this clinical indication. Its use was supported by numerous clinical studies that evaluated the safety and efficacy of this therapeutic agent published since 2009 and the pooled results of meta-analysis reporting that RTX was associated with improvement in skin score and stabilized lung involvement [6]. The real clinical benefits of RTX in SSc remained indeed contentious for a long time, given the differences in administered drug regimens, patient enrolment criteria, and follow-up duration of many uncontrolled trials. Nevertheless, two recently published randomized controlled trials confirmed the disease-modifying properties of RTX [7, 8], consistent with previously published observations.

The DesiReS trial was a Japanese double-blind, investigator-initiated, randomized, placebo-controlled trial that demonstrated the efficacy of RTX in reducing skin score at 24 weeks in patients with SSC. Further, a machine learning analysis of this trial showed that SSc patients with a high level of CD19^+^ B cells and a high baseline skin score exhibited the greatest improvement in skin involvement [9].

The RECITAL trial was a randomized, double-blind, phase 2b trial to assess the superiority of RTX compared with cyclophosphamide in patients with severe or progressive interstitial lung disease (ILD) related to SSc, idiopathic inflammatory myositis, or mixed connective tissue disease [8]. RTX was not superior to cyclophosphamide, although participants in both treatment groups had increased forced vital capacity at 24 weeks. Notably, RTX appeared to be better tolerated and associated with lower corticosteroid use than cyclophosphamide. The absence of superiority of RTX may reflect a better-than-expected outcome in the cyclophosphamide group rather than an absence of treatment response in the RTX group. It is important to highlight that during the follow-up, >85% of patients in both arms needed treatment with another immunosuppressive drug, underlining that it is necessary to treat aggressively patients with lung involvement and that also effective drugs such as RTX or cyclophosphamide may be inadequate as monotherapies.

Results of RECITAL and DesiReS, despite the differences in drug administration regimens (1000 mg at weeks 0 and 2 and 375/m^2^ once per week for 4 weeks, respectively), enrolment populations, and comparison with active treatment arm or placebo, confirmed that RTX is an effective treatment in SSc skin fibrosis and in patients with ILD related to connective tissue disease. The efficacy of RTX in apparently different diseases with similar organ involvement suggests that targeting B cells could represent an appropriate approach in a broad spectrum of connective tissue diseases with shared pathogenic pathways.

Indeed, these developments in SSc parallel the development of B cell targeted therapy in the other rheumatic diseases, rheumatoid arthritis, systemic lupus erythematosus, inflammatory myositis and ANCA vasculitis. In each case, compelling case series evidence has preceded, and often superseded, the more complex evidence from randomized trials [10, 11]. Further, in each case high-responder subpopulations and predictors of relapse have been identified from demographic, flow cytometric, genetic, gene expression and other immune biomarkers [12, 13].

Taken altogether, the evidence currently available suggests that the present therapeutic paradigm, to implement the treatment or employ immunosuppressants only when overt SSc clinical manifestations or organ damage ensue, is poised to evolve towards a molecular or biologically driven strategy to pre-empt tissue fibrosis, exploiting a ‘window of opportunity’ to treat patients with SSc before they developed organ damage.

In our opinion, the evidence from DesiReS and Recital and the evidence from the recent large trials of nintedanib in SSc-ILD [14] support the notion that a combination therapy consisting of immunosuppressant, B cell targeted approach and an anti-fibrotic drug could have an additive therapeutic effect in SSc patients.

## Data Availability

No new data were generated or analyzed in support of this article.
